# Immune biomarkers for head and neck cancer

**DOI:** 10.1007/s00262-025-04233-7

**Published:** 2025-12-18

**Authors:** Navid Sobhani, Alberto D’Angelo, Fernanda G. Kugeratski, Tristan Nguyen, Rachel M. Morris, Raheleh Roudi, Nikta Kia, Luca Pianta, Riccardo Morello, Stuart Winter, Daniele Generali, Alexandro Membrino

**Affiliations:** 1 Department of Leukemia, The University of Texas MD Anderson Cancer Center, Houston, TX USA; 2 Department of Medicine, Sheffield Teaching Hospital NHS, Foundation Trust, Sheffield, S57AT UK; 3 Department of Immunology, The University of Texas MD Anderson Cancer Center, Houston, TX 77030 USA; 4 Department of Cancer Biology, The University of Texas MD Anderson Cancer Center, Houston, TX 77030 USA; 5 Department of Radiology, Molecular Imaging Program at Stanford, Stanford University, Stanford, CA 94305 USA; 6 Acute and Critical Care Division, College of Nursing, University of Iowa, Lowa City, IA USA; 7 ENT Unit, ASST of Cremona, 26100 Cremona, Italy; 8 Nuffield Department of Surgical Sciences, University of Oxford, Oxford, UK; 9 Department of Medicine, Surgery and Health Sciences, University of Trieste, 34100 Trieste, Italy; 10 Department of Medicine (DMED), University of Udine, 33100 Udine, Italy; 11 Multidisciplinary Oncology ASST of Cremona, 26100 Cremona, Italy

**Keywords:** Head and neck cancer, Immune biomarkers, Immunotherapy, Stratification

## Abstract

In recent years, head and neck cancer therapy has been revolutionized by immunotherapy, which aims to restore the immune system’s anticancer activity suppressed by cancer cells. The major concern is that only a subset of patients responds to this therapy. Having specific biomarkers that could help identify the patients who are most likely to benefit from immunotherapies would be extremely useful. This review first explains the current immunotherapies tested in head and neck cancer, to then delve into the current prognostic and predictive biomarkers that have been investigated in this field to predict their responses. In our opinion, a broader selection of biomarkers could further improve who will respond best to immunotherapy.

**Supplementary Information:**

The online version contains supplementary material available at 10.1007/s00262-025-04233-7.

## Introduction

Head and neck squamous carcinoma (HNSCC) represents the predominant pathological subtype of head and neck cancer (HNC)[1] and ranks as the sixth most prevalent cancer globally and is a significant global health concern, with an estimated 880,000 new cases, and more than 450,000 deaths annually [2, 3]. Head and neck cancer survivorship imposes a significant economic burden on payers, healthcare systems, and patients, with median total costs of $372 exceeding baseline costs [4, 5].

HNSCC is generally characterized by an aggressive pattern of invasion, human papillomavirus (HPV) status as a key risk factor, and its immune profiling which may also serve as a predictor of patient outcomes [6]. This malignancy originates primarily from the epithelial tissues of the upper respiratory and digestive tract, encompassing the oral cavity, larynx and pharynx, with squamous cell carcinoma accounting for more than 90% of diagnoses [2, 3]. The etiology of HNSCC is multifactorial, with smoking tobacco and consuming alcohol ranking among the top risk factors, in conjunction with infection by high-risk HPV types, particularly HPV-16, which is highly correlated with oropharyngeal cancers [3, 7]. HPV status affects the disease process, as HPV status in HNC patients leads to different epidemic, genetic, and predictive or prognostic features. The last two elements are strictly related to HPV status, as they affect the immune landscape of the disease. HPV-positive HNCs occur more frequently in younger people with no or minimal history of alcohol or tobacco use. HPV-positive HNCs respond better to standard-of-care treatment, especially systemic treatment, regardless of the patient’s age [8].

Chronic inflammation, often induced by pathogenic microflora, exacerbates the genetic instability within epithelial cells, driving malignant transformation [3, 7]. In addition, the tumor microenvironment (TME), which can be characterized by hypoxia, immune suppression, and metabolic reprogramming, contributes to both tumor development and treatment failure [2, 9]. These TME interactions allow tumor cells to evade immune detection and create barriers to effective treatment [2, 7].

While conventional treatments have seen notable advancements, unfortunately they have yet to yield substantial improvement in the prognosis of advanced HNSCC. The 5-year survival rate has remained at 40% to 50% for decades, with disease recurrence or treatment failure occurring in nearly 30% of patients [2, 3, 7, 10]. This persistent challenge reflects the aggressive nature of HNSCC, its frequent metastasis to locoregional lymphatic structures, and the development of resistance mechanisms within the TME [3, 10]. Furthermore, the incidence of HNSCC is projected to increase by 40% over the next 16 years, underscoring the pressing need for treatments that are both innovative and effective [3].

Conventional therapies, while improving locoregional control, often fail to address the systemic and immunological aspects of HNSCC, necessitating a paradigm shift toward more integrative strategies [7, 10].

Immunotherapy has significantly reshaped treatment strategies for HNSCC. At present, it is particularly relevant to recurrent or metastatic disease, and research evaluating its role in de novo cases is ongoing. By targeting immune checkpoints regulating immune responses, immunotherapy aims to restore the antitumor activity suppressed by cancer cells. Immune checkpoint inhibitors (ICIs), including nivolumab and pembrolizumab, aim to reinvigorate antitumor immune responses that are suppressed by cancer cells by targeting key regulator pathways such as programmed cell death protein 1 (PD-1)/programmed death-ligand 1 (PD-L1), and cytotoxic T-lymphocyte-associated protein 4 (CTLA-4) [2, 7, 11]. These therapies have shown better outcomes in terms of overall survival (OS) and progression-free survival (PFS) relative to standard chemotherapy, with the greatest benefits observed in HNSCC cases with elevated levels of PD-L1 [7, 12]. However, just a subset of patients (~ 18%-20%) benefits significantly from them, highlighting the need for more precise approaches to fulfill the promise of precision medicine in cancer care, which aims to tailor treatments based on each patient's predicted response to targeted therapies, guided by specific biomarkers [13–15]. The response to treatment in cancer is crucial for assessing the safety and efficacy of new therapies and guiding clinical decision-making [16]. Treatment response refers to the measurable change in a patient’s cancer following therapy, typically evaluated by clinical endpoints [17] and can be assessed through parameters like OS, PFS [18], and objective response rate (ORR) [19].

Currently, researchers are exploring biomarkers such as the tumor mutational burden (TMB), interferon-gamma (IFN-γ) signatures, and tumor-infiltrating lymphocytes (TILs) in order to enhance the immunotherapy’s precision [2, 7, 20]. Additionally, innovative strategies that combine ICIs alongside chemotherapy, radiotherapy, or novel agents targeting the TME are currently being explored. Investigational approaches also include exosome-based therapies and dual checkpoint blockage, such as concurrent inhibition of PD-1 and CTLA-4, have shown promise in improving immune activation and overcoming resistance to therapy [2, 7]. The distinction between HPV-positive and HPV-negative HNSCC further emphasizes how important is to have tailored approaches to treat this cancer, as HPV-positive tumors often exhibit a more favorable immune profile than do HPV-negative ones [3, 21]. Unlike other HNSCCs, HPV-related cancers have better prognoses and OS than HNCs triggered by chemicals like alcohol and tobacco. The 5-year OS rate for HNC is barely 50% for chemical-triggered HNC. Even though HPV-positive patients with HNC have better prognoses than do HPV-negative ones, 10%-25% experience disease recurrence within 2 years of therapy [22]. Ang et al. showed in a clinical trial (NCT00047008) that HPV infection is a prognostic marker independent of other clinical variables in patients with oropharyngeal cancers. The study demonstrated better OS and PFS for HPV-positive than for HPV-negative patients (3-year OS rate, 82.4% vs. 57.1%, respectively) [23]. Another study conducted by Galvis et al. found that immunotherapy produced better OS (hazard ratio [HR], 0.77; p < 0.0001), comprising a longer survival (6.3 months-11.5 months) and better relative risk (1.29; *p* = 0.24) for HPV-positive patients than for HPV-negative patients. Furthermore, in a subgroup analysis, PD-L1-positive patients had longer OS than did those not expressing this marker (9.9 months vs. 6.5 months, respectively) [24].

**Fig. 1:**
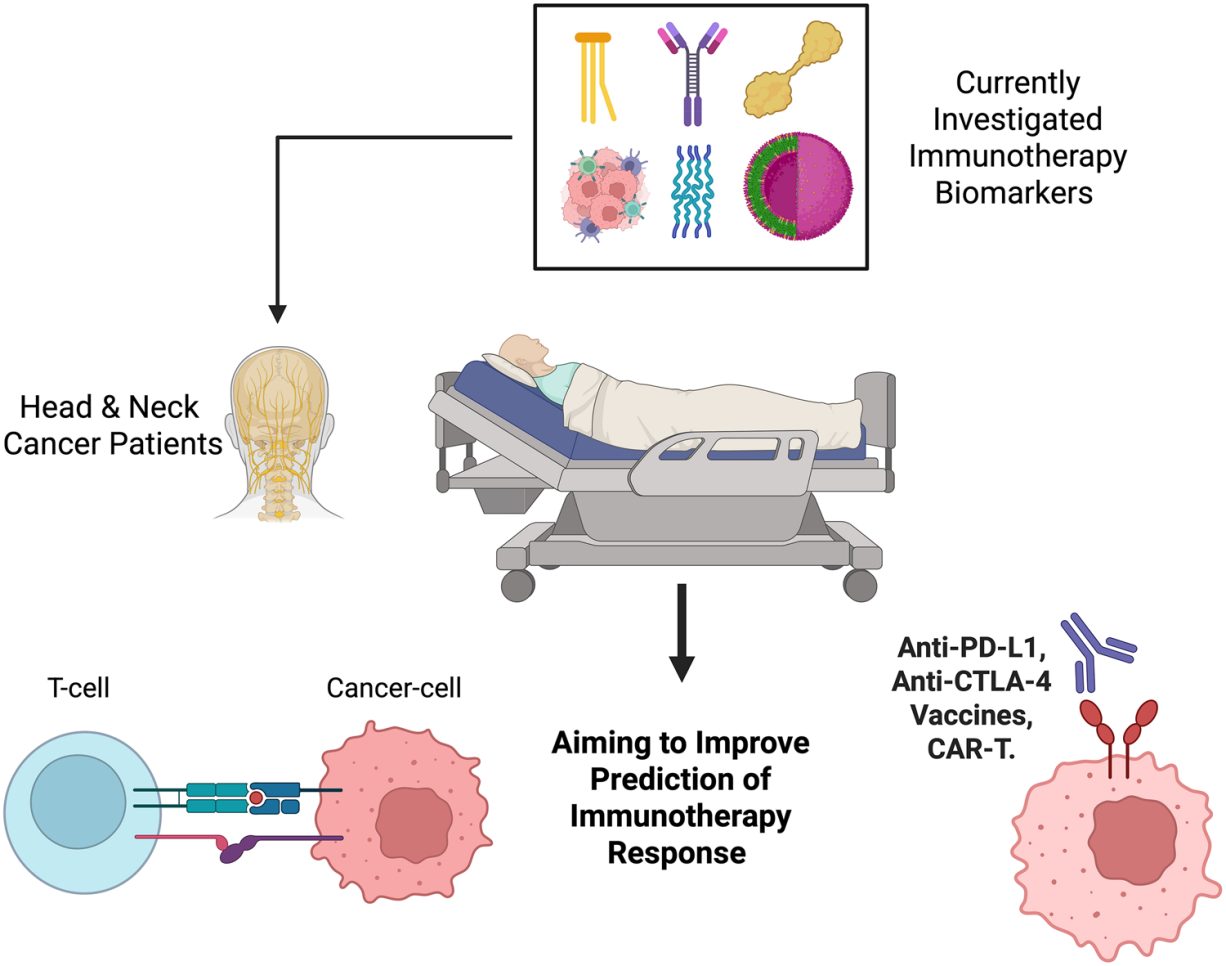
Immune biomarkers for head and neck cancer could be used to identify the patients most likely to respond to targeted therapies

Together, these emerging strategies collectively could reshape the therapeutic framework of HNSCC, fostering hope for sustained clinical benefit and better life quality (Fig. 1).

## ICI-based therapies

The introduction of ICIs into cancer treatment greatly improved HNSCC treatment. Treatment with ICIs evinced an improvement of the 1-year survival rates in recurrent metastatic patients from 36 to 57%, while the median survival duration from 7.7 to 13.0 months [25, 26]. Recent efforts have focused on optimizing ICI-based therapies for not only recurrent metastasis but also locally advanced HNSCC. Unleashing the immunity to kill cancer is a revolutionary strategy to cancer therapy and has been greatly beneficial against some cancer types (e.g., melanoma). The genomic alterations that characterize cancer can generate tumor antigens the immune system recognizes as foreign and illicit antitumor responses [25–28]. Common immunotherapeutic targets against HNSCC—such as the co-inhibitory receptors on T cells, which have important immunoregulatory functions—improve immunotherapy’s efficacy and overall antitumor response [29, 30]. T cells lose cannot target cancer cells when co-inhibitory receptors disrupt effector functions. These co-inhibitory receptors or immune checkpoints are upregulated after T cell receptor (TCR) signaling, co-stimulation, and inflammation activate T cells [31, 32].

In the section below, we describe some of the ICI-based therapies that block the specific inhibitory signals suppressing cell-mediated antitumor responses and prevent T cells from exerting their full effector functions against cancer cells, including HNSCC cells [33–37].

### PD-1/PD-L1 inhibitors

The adaptive immune system has evolved to eliminate threats from the body through the combined activities of B cells, CD4⁺ T cells, and CD8⁺ T cells. Upon antigen-mediated activation, which involves peptide–major histocompatibility complex engagement of the TCR and positive co-stimulatory signals initiated between CD28 on T cells and CD80 and/or CD86 (also known as B7.1 and B7.2, respectively) expressed on antigen-presenting cells (APCs), several negative regulators are expressed early and counteract positive signal activation. PD-1 ligands (PD-L1 and/or PD-L2) interact to disrupt positive signaling from the TCR and CD28 [33, 38–40]. PD-L1 level, TMB, and microsatellites are the only approved biomarkers for ICI responses [41].

In 2016, FDA approval was granted to two PD-1 inhibitors in patients with relapsed or metastatic HNSCC. These two monoclonal antibodies were pembrolizumab and nivolumab. Cemiplimab is an additional monoclonal antibody targeting PD-1 that has produced promising results in skin squamous cell carcinoma patients [42, 43]. Furthermore, atezolizumab and durvalumab are monoclonal antibodies targeting PD-L1.

Serving as a response biomarker, patient biomarkers disease status or clinical state are important to consider when selecting therapies against HNC. For example, Gavrielatou et al. [44] better survival rates following nivolumab treatment in patients with metastatic or recurrent HNSCC with high levels of stromal B cell infiltration than patients with low B cell stromal infiltration. Current research efforts are focused on maximizing ICI-based therapy’s potential and combining ICIs with conventional treatments. In a nonrandomized controlled trial, Saba et al. [45] showed that intensity-modulated reirradiation therapy plus nivolumab improved PFS in patients with recurrent or second-primary HNC. Also, a phase 3 study, whose follow-up was of 5 years (KEYNOTE-048), with a Japanese cohort demonstrated that treating recurrent and/or metastatic HNSCC with first-line pembrolizumab led to a better OS rate (30.4%) than did treating it with anti- EGFR drug cetuximab plus chemotherapy (10.5%) [46].

The safety of durvalumab or atezolizumab plus chemoradiotherapy in patients with HNSCC is being investigated. However, current anti-PD-1 or PD-L1 therapeutics have produced limited response [47–49].

Previous trials demonstrated that patients with recurrent or metastatic HNSCC treated with PD-1 inhibitors tolerated the drugs well, had ORR of up to 18%, and had long-lasting responses not usually observed with chemotherapy [50, 51]. PD-L1 expression levels could be used as biomarkers for anti-PD-L1 drugs. Therefore, PD-L1 is a static predictive biomarker for relapsed or metastatic HNSCC exhibiting a combined positive score (CPS) ≥ 1 [41]. Compared with standard-of-care chemotherapy, treatment of HNSCC with pembrolizumab or nivolumab resulted in better OS. However, researchers observed OR in less than 20% of patients, indicating the need for advancements in using ICIs to treat HNSCC [52, 53].

### CTLA-4 inhibitors

CTLA-4 is a transmembrane receptor found mainly in T cells. It is also mildly expressed in activated B cells, granulocytes, monocytes, and dendritic cells (DCs) [54]. CTLA-4 can inhibit APCs and is the antagonist of CD28 expressed on regulatory T cells. CTLA-4 present on regulatory T cells (Tregs) induces the immunosuppressive molecule TGFB after being activated by binding with transmembrane receptor CD28 binding [55]. CTLA-4’s binding to B7 protein induces T cell dysfunction and leads to negative immune response regulation. Both CTLA-4 and CD28 bind to the ligands CD80 and CD86, which are expressed on APCs. The affinity of CTLA-4 for CD80 and CD86 is higher than that for CD28 to CD80 and CD86. Therefore, in the presence of CTLA-4, CD28 does not bind to CD80 and CD86, and the immune system does not elicit an anticancer immune response [56].

Ipilimumab was the first CTLA-4 inhibitor approved by the FDA [57] followed by the approval of tremelimumab in 2022 [58]. In the IMCISION trial in patients with HNSCC comparing major pathological response (MPR) between treatment arms with nivolumab or nivolumab plus ipilimumab prior to extensive surgery, biomarkers such as higher PD-L1 expression, increased intratumoral CD8⁺ T cell infiltration, and the presence of APOBEC-associated mutations were all linked to MPR following neoadjuvant immunotherapy with nivolumab and ipilimumab [59]. In a phase 3 clinical investigation, the OS duration for melanoma patients receiving the CTLA-4 inhibitor tremelimumab was worse than that for patients receiving chemotherapy (10.7 months vs. 12.6 months, respectively) [60]. This disappointing result could have been due to the enrollment of patients with a favorable prognosis, demonstrating that the results in control patients were better than expected and that the immunotherapy scheduling was potentially suboptimal. However, in another study, combined treatment with regimens incorporating tremelimumab did not meet the primary endpoints for various solid tumors, including HNSCC [61]. The pathological response (PR) could be used as a surrogate to beneficial response to the ICI, anti-PD-1, as reported in trial NCT02641093 [62].

In the phase 3, randomized KESTREL trial, investigators recently compared durvalumab plus tremelimumab with the EXTREME regimen for HNSCC. The data demonstrated that, in HNSCC patients with high PD-L1 levels, durvalumab given alone or with tremelimumab was not superior to EXTREME in terms of OS. Notably, durvalumab administered alone or with tremelimumab produced longer response durations than did EXTREME (49.2% and 48.1% vs. 9.8%); however, the median PFS time was higher with EXTREME (2.8 months for both durvalumab groups vs. 5.4 months) [49]. Overall, for HNSCC, tremelimumab only moderately ameliorating effect when added to other therapies. This explains why FDA-approved anti-CTLA-4 therapies for HNSCC are lacking.

### Lymphocyte activation gene-3 inhibitors

One of the most promising ICIs include targeting lymphocyte activation gene-3 (LAG-3). This immune checkpoint protein co-localizes with the TCR and drives the Lck dissociation from CD4 and CD8 co-receptors through a conserved, repetitive glutamic acid–rich motif within the cytoplasmic tail of LAG-3, which reduces the pH at the immunological synapse and chelates the Zn2 + ions required to maintain Lck interaction with CD4 and CD8 [63]. However, the exact mechanisms by which LAG-3 regulates immune responses in vivo in specific contexts are not completely understood and likely will have to be elucidated to further enhance therapies targeting LAG-3 [21]. Both LAG-3 and PD-1 synergistically drive T cell exhaustion blocking the IFN-γ signaling required for tumor killing [64], and they both can be targeted to improve TIL infiltration. Enhanced PFS was demonstrated in melanoma patients treated with both relatlimab (an anti-LAG-3) and nivolumab (an anti-PD-1) compared with nivolumab alone [65, 66]. In 2022, treatment with relatlimab alongside nivolumab received FDA approval for melanoma at unresectable or metastatic state.

Some patients may not respond to PD-1 inhibitors due to inefficient infiltration of T cell into the TME. An analysis of tumor biopsies revealed that non-responders to therapy targeting PD-1 have few or no TILs. Strategies to enhance lymphocyte infiltration into tumors are being developed. One strategy involves stimulating APCs, such as DCs, to prime and activate T cells. APCs can be activated by soluble LAG-3, known for its binding affinity to MHC class II on APCs [67]. In the TACTI-002 trial, patients who had recurrent or metastatic HNSCC after chemotherapy were given eftilagimod alpha, a recombinant LAG-3 soluble protein, plus pembrolizumab. A complete response rate was observed in 13.5% of patients, with an ORR of 29.7%; these results were twofold higher than those for monotherapy with pembrolizumab (1.6% and 14.6%, respectively) and nivolumab (7.2% and 13.3%, respectively) [26, 53, 68].

### T cell immunoglobulin and mucin-domain containing-3 inhibitors

TIM-3 is a type I cell-surface glycoprotein. It is expressed on various tumor cells as well as immune subsets, such as CD4 T cells, CD8 T cells, Tils, Tregs and myeloid cells [69, 70]. TIM-3 interacts with its ligands GAL-9, CEACAM1, phosphatidylserine, and high-mobility group box 1 to induce T cell inhibition, and its expression in TILs is associated with cancer progression [71]. In esophageal squamous cell carcinoma patients, a higher level of PD-L1 and TIM-3 in TILs is correlated with poor OS [72]. Jie et al. [73] observed that in HNSCC patients, elevated levels of TIM-3 and PD-1 and in a subset of TILs that were CD8 + , were associated with poor clinical outcomes in those who received cetuximab. It has also been shown that TIM-3 expression in TILs correlated with increased tumor volume and metastasis to lymph nodes and that patients with HNSCC having TILs with low TIM-3 levels had improved prognoses and survival rates than did patients with high TIM-3 levels [74]. Blocking TIM-3 expression on T cells enhances proliferation, cytokine production, and immune activation [70]. Targeting TIM-3 plus PD-1 has shown the potential to enhance antitumor immunity and T cell function [75]. Anti-TIM-3 monoclonal antibodies and bispecific antibodies against both PD-1 and TIM-3 are being evaluated in clinical trials.

## Prognostic and immunotherapy response biomarkers

### Prognostic biomarkers

The most common viral infection in patients with HNSCC is cause by HPV-16, accounting for 90% of new cases. Additional viruses causing HNSCC are HPV-18, HPV-33, and HPV-35 [76].

The KEYNOTE-012 study demonstrated that HNSCC patients with HPV-positive status had better responses to ICIs than did HPV-negative patients [51]. In a central imaging review, the ORR in patients with HNSCC in the overall population was 18% (95% CI, 8%-32%), whereas HPV-positive patients had a higher ORR (25%; 95% CI, 7%-52%) than did HPV-negative patients (14%; 95% CI, 4%-32%) [51]. However, the phase 3 KEYNOTE-040 study did not support this finding, demonstrating no relationship between the status of HPV or PD-L1 levels with ICI-based treatment results.

In a study of patients with HNC, OR (per RECIST v1.1) was seen in 22% of cases, with a median response interval of 7.4 months (ranging between 2.8 and 45.8 months). This was associated with a median PFS duration of 2.6 months (ranging between 0.5 and 48.4 months) and a median OS duration of 6.0 months (ranging between 0.5 and 51.6 + months) [77]. Further studies demonstrated higher response rates in HPV-positive compared to HPV-negative patients after treatment with the PD-L1 inhibitor durvalumab [51] but no differences after treatment with atezolizumab [77].

Another infection commonly found in HNSCC patients is the Epstein–Barr virus (EBV) [78, 79]. EBV RNA has been linked with poor prognosis in these patients [79]. Nasopharyngeal carcinomas most commonly arise from the mucosal epithelium of the nasopharynx, with more than 70%-80% of new patients arising in East and Southeast Asia. Where EBV is endemic, it is responsible for carcinogenesis in the epithelium in up to 96% of all nasopharyngeal carcinoma cases. In these regions, screening programs could be implemented using liquid biopsy followed by nasal endoscopy and biopsy when needed. In fact, in patients with EBV-related HNCs, cell-free EBV DNA levels have been shown to be higher in cases with stage III-IV cancers than in those with stage I-II cancers [80, 81]. The response to systemic and local treatments can be confirmed by decreased circulating tumor EBV DNA levels.

Research has shown the potential role of circulating tumor DNA (ctDNA) utility in predicting treatment response in HNSCC patients including both HPV-positive and HPV-negative [82]. Reduction in ctDNA levels serves as a predictive marker of treatment response [83].

Local recurrence is easily detected by comparing current and prior magnetic resonance imaging and ctDNA results [84, 85]. These two diagnostic approaches are complementary, especially for nasopharyngeal carcinomas [86]. Also, EBV RNA could be a prognostic biomarker that should be tested in conjunction with ICI-based therapy.

Various studies have demonstrated a correlation between increased TIL numbers, especially for CD3 + and CD8 + cells, and improved outcomes in patients [87, 88]. In 2023, Li et al. reported that CD8 + T cells were significantly correlated with clinical outcomes in HNSCC patients, with the following top five mutated genes confirmed in a cohort in The Cancer Genome Atlas: CDKN2A, FAT1, MUC16, TP53, and TTN. High values of CD8 + T cells and stroma scores correlated with a higher probability of survival of HNSCC patients [89].

The TIL immunoscore (centered on the CD3/CD8, CD3/CD45RO, or CD8/CD45RO density) has emerged as a biomarker both in and around invasive tumors [90]. Therefore, it can be used to categorize patients with immunoscores of 0 to 4, proportionate to the densities in both regions. Some important studies demonstrated that patients with high to intermediate immunoscores have better survival than do those with low immunoscores [79, 91, 92]. The combination of CD8 + , Foxp3 + , and CD68 + cells in the immunoscore was shown to be a significant prognostic marker for HNSCCs [93–95]. Additionally, research focusing on CD8 + cytotoxic T cells, CD4 + helper T cells, and Foxp3 + Tregs in patients with HNSCC showed that TILs are associated with improved OS. Also, CD4 + T cell-rich tumors have been shown to be better therapies for immunotherapy and to correlate with better OS than CD4 + cell-depleted cancers [96]. Furthermore, CD3 + T cells correlated with improved OS after HNSCC therapy [97]. Finally, in humans, cytotoxic CD8 + T cells alongside tissue-resident memory (TRM) T cells have been shown to play important immune-protective roles, promote antitumor immunity, and correlate with improved HNSCC survival [98, 99]. TRM T cells are enriched with PD-L1 and LAG-3 and expand notably during ICI-based therapy. Additionally, CD8 + CD103 + tissue-resident memory T cells correlate with better survival, suggesting an important role for these cells against solid tumors, including HNSCC, using ICIs [98, 100].

Other notable prognostic biomarkers for HNSCC are mutations of the TP53, NOTCH1, and CDKN2A genes, which have been associated with worse OS in HNSCC patients [101]. Another very important emerging biomarker for HNSCC is ctDNA, detected using liquid biopsy.

Cell-Free DNA (cfDNA) can be found in both patients and controls. A liquid biopsy should be based on highly sensitive techniques able to distinguish ctDNA and cfDNA. cfDNA refers to fragmented DNA released into the bloodstream from both normal and malignant cells, whereas ctDNA is the tumor-derived fraction of cfDNA. Many biases can affect a liquid biopsy sensitivity and specificity. For example, cfDNA can increase with age in either patients (p = 0.000002) or healthy individuals (p = 0.04) [102]. In fact, aging can be considered a chronic condition characterized by low-grade inflammation in which both metabolites and nucleic acids accumulate in the bloodstream due to less-efficient cellular systems for DNA degradation [102]. In head and neck cancer, cfDNA levels are elevated compared with healthy individuals and show strong associations with advanced stage, nodal involvement, and recurrence risk. Levels often decline after tumor resection or treatment, but persistent or rising cfDNA predicts poorer progression-free and overall survival. While cfDNA provides a useful measure of overall tumor burden, its limited specificity highlights the added value of ctDNA, which offers tumor-specific genomic and viral information for more precise disease monitoring [102–104]. One study demonstrated that ctDNA negativity correlated with markedly better prognosis than did ctDNA positivity in patients with HNSCC [105]. In another study, ctDNA was present in 5/7 (71.4%) of the total recurrent patients. Honoré et al. [106] showed that patients with detectable ctDNA after treatment had significantly worse RFS and OS. Hanna et al. [107] found that ctDNA positivity indicates disease progression and is associated with inferior survival. Therefore, ctDNA may serve as a prognostic biomarker for HNSCC too [108].

### Immune biomarkers

**Fig. 2:**
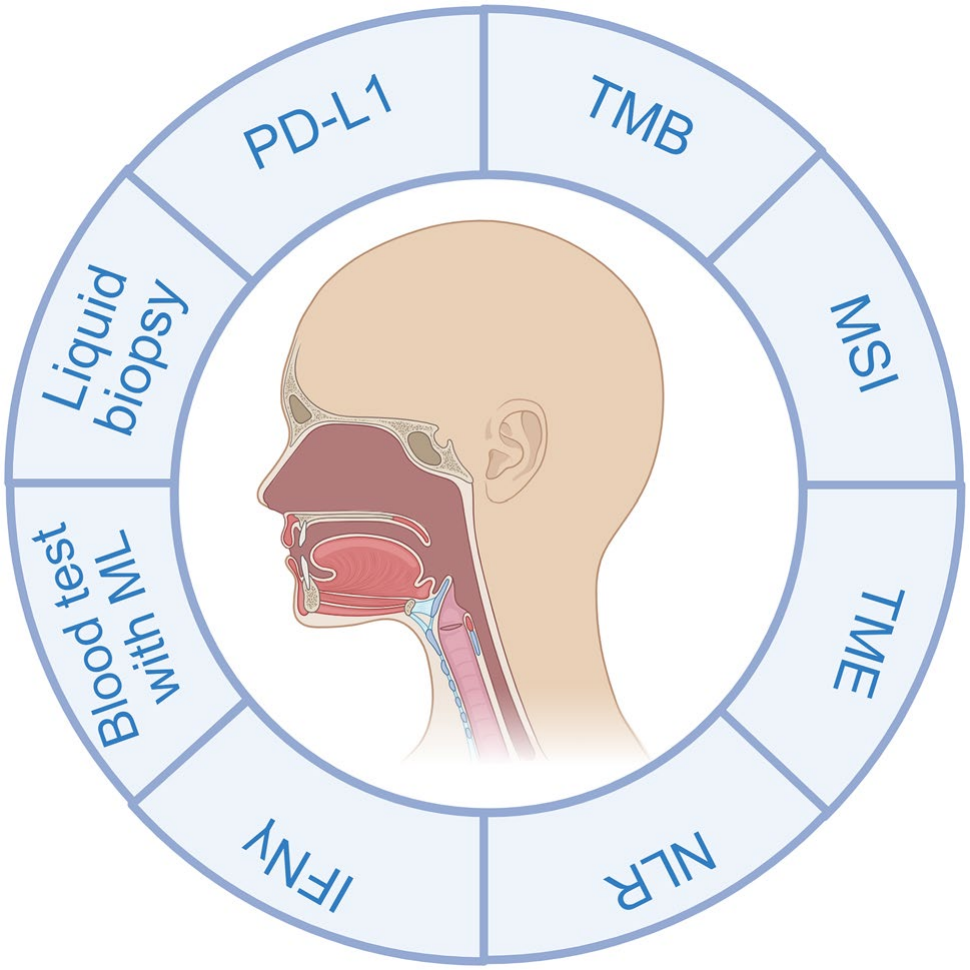
Biomarkers for assessment of the ICI response of HNCs. MSI, microsatellite instability; ML, machine learning; and NLR, neutrophil-to-lymphocyte ratio

Checkpoint inhibitors are the mainstream immunotherapeutics being tested for HNC and biomarkers. However, most patients do not have responses to them and experience toxic effects [109, 110]. Efficient, reliable predictive and prognostic biomarkers are critical in optimizing patient selection (Fig. 2). Selecting the right patients maximizes positive therapeutic outcomes and improves overall patient care.

#### PD-L1

Pretreatment levels of PD-L1 on immune cells in tumor biopsies has demonstrated antigen-specific responses [111] and correlated with improved therapy outcomes [112]. Tumor cells become sensitive to ICIs when they overexpress PD-L1, inhibiting the immune response. The gene for PD-L1 is found on chromosome 9p, which also hosts another fundamental element of immune escape used by tumor cells: the IFN-γ signaling pathway coding regions. If mutations appear in these sequences, the immune system cannot recognize tumor cells on its own [113]. In 2019, Chen et al. [114] showed that p16 protein expression correlated with PD-L1 expression in HNC cases, suggesting that patients with high levels of PD-L1 may benefit from treatment directed against PD-1/PD-L1. Tumors expressing PD-L1 usually respond best to these agents [109].

Compared to HPV-negative HNCs, HPV-positive HNCs have higher numbers of NK cells, T cells, granulocyte cells, and TIL-B cells and reduced numbers of Tregs, fibroblasts, and macrophages. This results from the expression of E6 and E7 on cell surfaces, which triggers cytotoxic T-lymphocyte infiltration. Therapeutic approaches to HNC can be based on HPV status, and immunohistochemical staining for p16 expression serves as a reliable surrogate marker for HPV detection in HNSCC. Researchers have shown that this staining (both nuclear and cytoplasmic in more than 70% of cells) can indicate consistent HPV infection and allow for HPV-specific testing of tumor samples. If immunohistochemistry results are positive for HPV, in situ hybridization assays can detect viral DNA and RNA. Specifically, DNA in situ hybridization reveals integrated HPV DNA, whereas RNA in situ hybridization detects actively transcribed HPV DNA [115].

Tissue biopsy samples are usually processed as formalin-fixed, paraffin-embedded samples, but this sample-preparation method presents challenges regarding DNA and RNA in situ hybridization, as it preserves the morphology and cellular details of a tumor but inhibits real-time quantitative polymerase chain reaction (PCR) for nucleic acids extracted from these samples. Fresh frozen samples should be considered for such assays, which are costly but timesaving [115].

The CPS, which quantifies PD-L1 expression in macrophages and lymphocytes in relation to the total tumor cell numbers, guides clinicians regarding treatment options for diseases such as HNSCC. PD-L1, a protein expressed inside the TME, is usually evaluated using immunohistochemistry. PD-L1 level can also be presented as the tumor proportion score (TPS). The CPS and TPS are calculated differently. The TPS measures the number of neoplastic cells with either complete or partial PD-L1 staining. The CPS quantifies PD-L1 expression by taking the sum of PD-L1 staining tumor and immune cells, dividing it by the total count of viable tumor cells, and multiplying the result by 100 [116]. In KEYNOTE-012, a phase 1b clinical trial of 188 HNSCC patients, a CPS of 1 or higher was considered a significant predictor of HNSCC’s response to pembrolizumab. CPS-positive patients accounted for 81% of the cohort and had better ORRs, PFS, and OS than CPS-negative patients. In contrast, TPS-positive patients, who accounted for 65% of the patients, did not have such a correlation [50, 117].

Various CPS cutoffs have been used by different institutions, which has changed the predictive outcomes. In one study, the use of various CPS cutoffs produced different statistical outcomes, such as for PFS and ORR. For example, the use of the 22C3 diagnostic platform with a CPS cutoff of 2% demonstrated that the ORR was marginally greater in PD-L1-positive tumor cases (18%) than in PD-L1-negative tumor cases (12%). Also, the PFS rate using a 1% CPS cutoff was substantially higher for PD-L1-positive patients (24% for CPS ≥ 1%, 31% for CPS ≥ 50%) than for PD-L1-negative patients (20% for both CPS ≤ 1% and CPS ≤ 50%) [118]. Raising the cutoff increased the chances of predicting a higher percentage of patients with favorable PFS. Importantly, the CPS was still a very sensitive survival predictor, even at the 1% cutoff. Consequently, following the findings of the randomized phase 3 KEYNOTE-040 trial demonstrating that the CPS demonstrated greater sensitivity than TPS at lower threshold values in predicting pembrolizumab efficacy, CPS developed as the preferred scoring method for evaluating PD-L1 expression HNSCC [119]. In KEYNOTE-040, HNSCC patients treated with pembrolizumab had a median OS time of 8.7 months when the CPS was 1 or higher vs. 6.3 months when it was less than 1, demonstrating that the CPS could identify patients with better outcomes.

Additionally, the superiority of the CPS to the TPS was shown in the phase 3 KEYNOTE-048 trial. The outcome of the trial supports the use of CPS as a biomarker for HNSCC [120, 121]. This randomized clinical trial involving 882 patients with incurable metastatic HNSCC tested pembrolizumab as single therapy, pembrolizumab alongside chemotherapy, and cetuximab alongside chemotherapy (the EXTREME scheme). In the CPS 20 or higher (OS HR: 0.61; 95% CI [0.45, 0.83]; p = 0.0007) and CPS 1 or higher (OS HR: 0.78; 95% CI [0.64, 0.96]; p = 0.0086) groups, pembrolizumab significantly enhanced OS compared to cetuximab combined with chemotherapy [122]. Accordingly, the anti-PD-1 antibodies pembrolizumab and nivolumab received FDA approval to treat HNSCC recurring after the use of platinum-based regimens. Pembrolizumab also was approved alongside chemotherapy (platinum and fluorouracil) for all HNSCC patients and received approval as a single-agent therapy for patients PD-L1-positive patients with CPS ≥ 1 [123]. These data demonstrated the importance of PD-L1 as a predictive biomarker for the response of solid tumors to anti-PD-L1 therapies [124]. Additionally, PD-L1 expression associated with better clinical outcomes in the phase 3 randomized CheckMate 141 trial testing nivolumab in post-platinum therapy for HNSCC. In fact, a 2-year follow-up study of patients enrolled in the CheckMate 141 trial demonstrated that nivolumab use improved OS more than treatment with investigator-chosen drugs (HR, 0.68; 95% CI, 0.54–0.86). The OS rate for the nivolumab-treated patients was three times higher than that in the investigator-chosen-drugs group (16.9% vs. 6.0%), demonstrating an OS benefit for patients with PD-L1 TPS ≥ 1% (HR, 0.55; 95% CI, 0.39–0.78) and those with a TPS < 1% (HR, 0.73; 95% CI, 0.49–1.09), independently from the HPV status [125]. Keynote-689 Phase III randomized clinical investigation made of 714 cases tested perioperative pembrolizumab with SOC vs SOC alone in resectable HNSCC patients. Adding pembrolizumab to SOC prevented cancer recurrence for 5 years. In the CPS ≥ 10 group: HR 0.66, 95% CI 0.49–0.88, P = 0.00217, whereas in the CPS ≥ 1 group: HR 0.70, 95% CI 0.55–0.89, P = 0.00140, in all patients [126]. The data indicate that changing the cutoff from CPS 1 to CPS 10 does not significantly impact the HR.

An intriguing issue has been determination of PD-L1 expression based on the cutoffs defining positivity of expression and the type of experiment conducted [123]. Various studies demonstrated cutoff variations ranging from 1 to 50%, making comparisons very challenging [121, 127]. Interestingly, for breast cancer, especially triple negative breast cancer, a CPS ≥ 10 justifies the use of immunotherapy, based on the PD-L1 FDA-approved test IHC 22C3 pharmDx assay, developed by Dako (a division of Agilent Technologies)[128]. For HNSCC that is recurring or metastatic, a CPS ≥ 1 is the FDA-approved threshold that justifies the using pembrolizumab in the first-line setting either as single agent, or alongside chemotherapy. This is following the findings from KEYNOTE-048 trial [120, 121].

Moreover, we conducted our meta-analysis incorporating all the clinical trials available on PubMed until August 2025, examining different CPS or TPS cutoff values and response to therapy directed against PD-L1/PD-1. We noticed that in the group that was using CPS or TPS cutoff ≥ 1, the OR was 0.73 [95% CI: 0.64, 0.82]. The value did not change significantly from the group with CPS with cutoff ≥ 10, which has an OR: 0.64; [95% CI: 0.51, 0.79] (Fig. S1). Although more clinical investigations should be conducted in prospective clinical trials for larger CPS that would be beneficial to confirm this data, we do not see any significant benefits in increasing from CPS ≥ 1 to CPS ≥ 10.

#### Tumor mutational burden

Neoepitopes on the surface of tumor cells are generated through genetic mutations. Additionally, a high missense mutation load increases CD8 + T cell density and improves clinical outcomes. RNA sequencing across various tumors has demonstrated that specific immunogenic mutations correlate with improved survival outcomes and linked to elevated CD8A expression alongside markers for immune-exhaustion marker levels, making response to immunotherapy more likely.

The TMB, often expressed as the total count of mutations per exome or of mutations per mega-base of sequenced DNA, measures the burden of coding mutations across the tumor genome. The higher the TMB, the higher the number of neoantigens the tumor produces and the greater the likelihood of triggering strong antitumor immunity and immune evasion, which is often correlated with enhanced ICI sensitivity. In HNC, the high TMB range is generally 10%-19% [129, 130]. The TMB is a potential biomarker for predicting immunotherapy efficacy [131]. A meta-analysis of 1770 patients across 12 clinical trials, comprising those with solid tumors treated with pembrolizumab (235 HNSCC patients), demonstrated how high TMBs are associated with significantly improved ORR and OS, independent of PD-L1 expression [132]. In 2022, Haddad et al. [133] demonstrated that higher TMB was predictive of better OR, greater neoantigen load, and favorable clinical response to pembrolizumab in HNSCC advanced cases. In the KEYNOTE-012 trial, the TMB cutoff was set at 102% or higher, which positively correlated with the response to immunotherapy [51, 134]. In another cohort of 126 HNC patients, among those receiving therapy against PD-1/PD-L1, a high TMB was a positive predictor of the response to immunotherapy in patients from the same group with HPV-negative/EBV-negative tumors [135].

A meta-analysis comprised of 1200 HNSCC patients treated with ICIs demonstrated an association between a high TMB and considerably ameliorated OS (odds ratio, 2.62; 95% CI, 1.74–3.94]; p < 0.0001) and overall advantage in survival (HR, 0.53; 95% CI, 0.39–0.71; p < 0.0001) [136]. A newer clinical trial involving 674 patients across eight types of cancers, comprising HNSCC, found that individuals with a high TMB had OS significantly improved (HR, 0.61; UCB, 0.84; p = 0.005), PFS (HR, 0.62; UCB, 0.82; p = 0.003), and time to progression (TTP) (HR, 0.67; UCB, 0.92; p = 0.02) against TMB. Moreover, a high TMB was significantly correlated with improved OS independent of the ICI used (pembrolizumab: HR, 0.67; UCB, 0.94; p = 0.03; other ICIs: HR, 0.37; UCB, 0.85; p = 0.03) [137]. On the other hand, two studies analyzing The Cancer Genome Atlas and Chicago Head and Neck Genomics data did not demonstrate a correlation between the TMB and immune cell infiltrates [123, 138, 139]. This probably explains why the FDA has not approved the TMB as an immunotherapy biomarker for HNSCC [123].

There is a major drawback of using the TMB as a predictive HNSCC immunotherapy response biomarker. The occurrence of multiple tumor subclones, which generate heterogeneous neoantigen landscape that may impact host immune recognition, even with a TMB. These subclones could be a reason why TMB does not respond to ICIs [140]. Another issue is the lack of consensus methods that could optimally identify TMB cutoffs. Results from a meta-analysis comprised of 11 investigations, looking at TMB cutoff values demonstrated that they ranged from 6 to 175 mutations/exome (mean, 130 mutations/exome). This study demonstrated how large these variations can be. Results also depend on the type of sequencing methods used. In the past, the NGS was considered as the gold standard for TMB measurement. Currently, WES is the standard for TMB measurement [141, 142].

#### Microsatellite instability

Deficiencies in the DNA mismatch repair (MMR) lead directly to microsatellite instability (MSI). MMR deficiencies lead to the accrual of mutations in small pieces of DNA, called microsatellites, that repeat along the genome. Tumorigenesis is driven by the accumulation of these mutations, which are responsible for neoantigen expression and the enhancement of the immune response to these neoantigens [143]. High MSI (MSI-H) arises from the loss of function of MLH1, MSH2, MSH6, or PMS2, often due to MLH1-promoter methylation. PCR and NGS can be used to assess MSI-H status, which strongly correlates with the expression levels of the respective MMR proteins [144]. In one study, MSI in 126 patients with HNSCC was higher among responders to anti-PD-1/PD-L1 therapy than among non-responders [135].

Recently, MMR scarcity and MSI were shown to anticipate the immunotherapy response in various types of solid tumors. In 2015 and 2017, Le and colleagues found that pembrolizumab produced a strong, objective radiographic response in 40% to 53% of MMR-deficient tumors patients initiated from various organs, but not in patients with MMR- containing tumors [121, 145]. The data from five clinical trials—with a total of 149 patients—led to approval by the FDA of pembrolizumab for unresectable MSI-H treatment of or MMR-deficient solid tumors [146]. Data from the phase 2 KEYNOTE-158 trial demonstrated that PD-1 blockage produced promising results against MSI-H or MMR-deficient cancer in many different types of solid tumors [147]. This trial enrolled 233 cases encompassing 27 different types of solid cancers who had undergone treatment and had known MSI status. Following a median follow-up of 13.4 months, the ORR was 34.3% (95% CI, 28.3–40.8%), with a median PFS duration of 4.1 months (95% CI, 2.4–4.9 months) and median OS duration of 23.5 months (95% CI, from 13.5 months to not reached)[147].

Not all MMR-deficient cancers respond to ICIs, suggesting that other factors contribute to the response. These factors include MSI intensity, changes in antigen presentation, inadequate T cell activation, T cell exhaustion, and other immune system actors like macrophages, natural killer cells, and neutrophils, which may affect the adequacy of the response to ICIs [148].

The neutrophil-to-lymphocyte ratio (NLR), an easily obtainable marker of systemic inflammation, has been shown to carry prognostic value in cancer. A high NLR reflects increased neutrophil-mediated tumor-promoting inflammation coupled with reduced lymphocyte-mediated antitumor immunity. Multiple studies have demonstrated that an elevated NLR is associated with poorer survival outcomes in HNSCC patients [149]. Similar findings have been reported across a range of malignancies, including colorectal, gastric, lung, and breast cancers, where higher NLR consistently correlates with advanced disease stage, treatment resistance, and inferior overall survival [150, 151]. These data support the incorporation of NLR as a broadly applicable, cost-effective prognostic biomarker in oncology. A clinically useful parameter is the NLR. It may reflect the balance between inflammation-mediated immunity, which favors tumor growth-suppressing or tumor growth-silencing T cells, and antitumor immunity. The higher the NLR, the worse the prognosis [152, 153] (Table 1).

**Table 1:** Ongoing Clinical Trials for Biomarkers of Immunotherapy Response of HNC

Trial identifier	Investigation plan	Treatment	Clinical setting	Biomarkers of response	Phase	Trial status
NCT05328024	Case-only, samples with DNA	Immunotherapy	Prospective	Anti-PD-1 biomarkers	N/A	Recruiting
NCT05375266	Noninterventional	Standard sampling	Prospective	Immunological biomarkers	N/A	Recruiting
NCT06163534	Noninterventional	Standard sampling	Prospective	ctDNA, NGS, RNA biomarkers	N/A	Recruiting
NCT04146064	Noninterventional	Breathing sampling	Prospective	Immunotherapy biomarkers	N/A	Recruiting
NCT06706401	Randomized, factorial assignment, open label	Tretinoin (Vesanoid), radiotherapy, cisplatin, cetuximab	First line	Immunotherapy biomarkers	3	Not yet recruiting
NCT05025813	Nonrandomized, single-arm assignment, open label	Pembrolizumab	First line	Immunotherapy biomarkers	2	Recruiting
NCT06084897	Randomized, parallel assignment, open label	Anti-PD-1, chemotherapy, radiotherapy	First line	Immunotherapy biomarkers	2	Recruiting
NCT05704985	Nonrandomized, sequential assignment, open label	DK210, radiotherapy, ICI, chemotherapy	First line	Immunotherapy biomarkers	1	Recruiting
NCT05047094	Nonrandomized, single-arm assignment, open label	Pembrolizumab, radiotherapy	First line	Immunotherapy biomarkers (including CD3, CD4, CD8, CD69, and CD137)	N/A	Recruiting
NCT06319963	Nonrandomized, sequential assignment, open label	Lenti-HPV-07	Second line	T cell activation/effector/memory marker	1/2	Recruiting
NCT06385730	Nonrandomized, parallel assignment, open label	Anti-PD-1, radiotherapy	First line	Immunotherapy biomarkers	2	Recruiting
NCT06601309	Nonrandomized, single group assignment, open label	Serplulimab, paclitaxel + cisplatin, radiotherapy	First line	Immunotherapy biomarkers	2	Recruiting
NCT06178211	Nonrandomized, single group assignment, open label	Adebrelimab, carboplatin + nab-paclitaxel	First line	ctDNA or PD-L1 expression	2	Not yet recruiting
NCT06321640	Noninterventional	Sampling	Prospective	Immunotherapy biomarkers	N/A	Recruiting
NCT04671667	Randomized, parallel assignment, open label	Carboplatin, cisplatin, CT, radiotherapy, MRI, radiotherapy, pembrolizumab	First line	PD-L1 expression	2	Recruiting
NCT01810913	Randomized, parallel assignment, open label	Atezolizumab	First line	Immunotherapy biomarkers	2/3	Recruiting
NCT05520099	Case-only, samples with DNA	Fresh tissue sampling	Prospective	PD-L1 expression and TMB	N/A	Recruiting
NCT05323890	Nonrandomized, single group assignment, open label	Tislelizumab, chemotherapy, radiotherapy	Prospective	Immunotherapy biomarkers	2	Recruiting
NCT04815720	Nonrandomized, single group assignment, open label	Pepinemab, pembrolizumab	First line	Immunotherapy biomarkers	1/2	Recruiting
NCT05286294	Nonrandomized, single group assignment, open label	Fecal microbiota transplant	First line	Immunotherapy biomarkers	2	Recruiting
NCT06272214	Randomized, parallel assignment, open label	Radiotherapy, immunotherapy	Second line	Immunotherapy biomarkers	2	Recruiting
NCT06498752	Nonrandomized, parallel assignment, open label	Anti-PD-1	First line	ctDNA	2	Recruiting
NCT05743504	Nonrandomized, single group assignment, open label	Atezolizumab	First line	Immune-related biomarkers, VEGF-A, transforming growth factor-β1, soluble PD-L1	1/2	Recruiting
NCT05267626	Nonrandomized, sequential assignment, open label	AU-007, aldesleukin, avelumab	First line	Immune-related biomarkers	1/2	Recruiting
NCT06029270	Randomized, sequential assignment, open label	Relatlimab, immunotherapy	First line	Postinduction EBV DNA as a predictive biomarker	2	Recruiting
NCT06675201	Randomized, sequential assignment, open label	Neoantigen-loaded dendritic cells	Second line	TMB, PD-L1, and ctDNA	2	Recruiting
NCT06415669	Nonrandomized, single group assignment, open label	Paclitaxel, adebrelimab, apatinib mesylate	Second line	TPS	2	Recruiting

CT, computed tomography; MRI, magnetic resonance imaging; and VEGF-A, vascular endothelial growth factor A

#### Tumor-infiltrating cells

Tumors engage in a dynamic interplay with the surrounding microenvironment. The immune cells within the TME can predict the response to immunotherapy [154]. Strong evidence suggests that, during tumorigenesis, the immune system evolves into a state of pro-inhibitory activity, increasing anti-inflammatory cells and decreasing antitumor cells. This process explains how a strong inflammatory insult, such as surgical intervention, may accelerate the spread of a tumor that was undetectable via computed tomography, as inflammation promotes tumor proliferation, metastasis, and the destruction of adaptive immunity.

ICIs are less effective against tumors with less CD8 + T cell clones and no CD4 + T cell stimuli supporting the immune system’s antitumor activity. IFN-γ is expressed by natural killer cells to stimulate T cells to kill tumor cells, and patients with low natural killer cell numbers in the peripheral blood have less of a response to ICIs. Another biomarker correlated with the immune system is the presence of B cells in tumor tissue, representing a tertiary lymphoid structure (TLS) with enhanced response to ICIs. TLS is ectopic lymphoid aggregates that resemble secondary lymphoid organs and develop within non-lymphoid tissues in response to chronic inflammation, including cancer. TLS are typically composed of B cell follicles, T cell zones with mature dendritic cells, and high endothelial venules, supporting local antigen presentation and adaptive immune priming [155]. In solid tumors, the presence of TLS has been correlated with improved clinical outcomes and enhanced response to immune checkpoint blockade, as demonstrated in melanoma, renal cell carcinoma, and lung cancer [156, 157]. In HNSCC specifically, TLS-like aggregates enriched in B and T cells have been associated with favorable prognosis and better response to immunotherapy [158]. These findings highlight TLS as an emerging biomarker reflecting the quality of local antitumor immunity across solid malignancies, including HNC. Antigen-specific T cells against tumors are created if tumor antigens are presented by DCs to draining lymph nodes. Thus, tumors with high numbers of DCs infiltrates are responsible for an increased likelihood of tumor response to ICIs, as the immune system can recognize new antigens [159].

In patients with HNSCC treated with ICIs, increased densities of tumor-infiltrating immune cells including T cells, APCs, and NKs have been associated with improved ORR [160]. In 2018, Kim et al. [161] observed that a high CD8 + lymphocyte rate, together with PD-1 expression, co-stains with PD-L1. The authors also observed that frameshift events in ICIs among patients with HNSCC were higher in responders than in non-responders.

Tumor immune-infiltrating cells, particularly CD56 + natural killer cells, correlated with improved responses in patients with HNSCC treated with ICIs [135]. More evidence about the role of tumor immune-infiltrating cells in HNSCC and a more standardized method of detecting these cells before they can be fully exploited is needed for the prediction of the immunotherapy response for this type of cancer.

An analysis of B cell infiltration in tissue samples from 60 patients with platinum-refractory HNSCC before and after treatment with nivolumab demonstrated that a high pretreatment density of B cells correlated with extended PFS [44]. Because B cells and TLSs are known to promote the ICI response [157], researchers evaluated whether TLSs are linked with improved survival. In a cohort of HNSCC patients (38 TLS- and 12 TLS +), investigators did not observe a significant association of TLS with PFS or OS, which can likely be attributed to the reduced number of patients in the TLS + cohort [44]. Another study demonstrated that TLSs in the HNSCC TME can be a prognostic biomarker for improved survival and a predictive biomarker for improved responses to ICIs [162].

## Discussion

HNSCC incidence is projected to rise by 30% in the next five years, largely due to increasing HPV infections [8]. Oropharyngeal caused by HPS is more prevalent in younger individuals with minimal exposure to tobacco or alcohol [23]. Public health efforts, including HPV vaccination and lifestyle interventions, aim to curb this trend. Early detection remains essential for improving outcomes [22], and HPV-positive patients generally respond better to ICIs [23]. Beyond HPV, other etiological factors include tobacco, alcohol, poor diet, environmental exposures, and socioeconomic disparities [8]. These contribute to carcinogenesis through epigenetic changes and limited access to screening.

ICIs have significantly improved HNSCC treatment outcomes. Clinical trials, such as NCT00047008 and Keynote-689, demonstrated increased survival rates and reduced recurrence when ICIs like pembrolizumab were added to standard care [25, 26, 44]. Several biomarkers are being investigated to predict ICI response, comprising PD-L1 expression, TMB, MSI, TME features, NLR, and IFN-γ signatures [41, 51, 102, 163].

NLR and IFN-γ are not validated biomarkers for personalized therapy in HNSCC, but rather indicators of immune dysfunction and effector activity, respectively. Elevated NLR has been linked to poor outcomes in HNSCC and other cancers [164, 165], while IFN-γ has been associated with favorable immunotherapy responses in several malignancies [166, 167]. Although not yet actionable in the clinic, these markers highlight the potential of immune-related parameters to inform future patient stratification.

Liquid biopsy techniques offer analysis of some useful plasma-derived molecules, such as ctDNA and extracellular vesicles (EVs), that can serve as biomarkers to predict treatment response [102, 168]. Circulating tumor EBV DNA as a specific type of ctDNA biomarker [169] and EVs found in bodily fluids have both shown potential as biomarkers of treatment response [170]. EVs are nanosized, membrane-bound vesicles released by nearly all cell types, including tumor and immune cells. They are broadly categorized into exosomes (30–150 nm) and microvesicles (100–1000 nm), which differ in their biogenesis but share overlapping cargo consisting of proteins, lipids, DNA, and RNA species that mediate intercellular communication [171]. In HNCs, tumor-derived EVs contribute to tumor progression by promoting immune evasion, angiogenesis, and metastatic spread [172]. Importantly, EVs isolated from the blood, saliva, or other body fluids of patients with HNCs have been shown to carry tumor-specific molecular signatures, including PD-L1, EGFR, and non-coding RNAs, which correlate with disease stage and treatment response [173, 174]. These findings highlight EVs as a promising class of non-invasive biomarkers for early detection, prognosis, and therapy monitoring in HNCs.

PD-L1 remains a key predictive biomarker, but inconsistencies in testing methods and thresholds hinder its clinical utility [42, 43, 175]. Recent evidence suggests that analyzing PD-L1 at relapse may yield more accurate predictions than using archival samples. Despite progress, challenges persist. For example, durvalumab and tremelimumab combination could not gain FDA approval due to patient selection biases [147]. Tissue biopsies may not reflect tumor heterogeneity, making liquid biopsy a promising alternative [123, 147]. Tumor evolution through treatment further complicates ICI efficacy, reinforcing the need for ongoing molecular response monitoring [51, 163].

Emerging biomarkers like NLR and IFN-γ offer additional avenues for personalized therapy. ctDNA is particularly useful for assessing molecular residual disease, enabling earlier intervention and reducing unnecessary treatment [41]. However, single-time-point biomarker assessments are insufficient due to tumor evolution; repeated testing is necessary to capture dynamic changes. Moreover, machine learning tools, such as SCORPIO, show promise in integrating clinical and molecular data to optimize treatment strategies [163]. These approaches could enhance therapeutic sequencing, reduce toxicity, and improve survival and quality of life.

Currently, HPV status and PD-L1 CPS remain the most widely established biomarkers with direct implications for clinical decision-making in head and neck cancer. ctDNA is an emerging biomarker that is under active clinical evaluation and has shown promise as a minimally invasive tool for disease monitoring and treatment guidance. In contrast, immune-related markers such as chemokines and IFN-γ have largely been reported in retrospective studies, where they demonstrate prognostic or predictive associations, but they are not yet incorporated into standard clinical practice.

## Supplementary Information

Below is the link to the electronic supplementary material.

Supplementary file1 (DOCX 3858 KB)

## Data Availability

No datasets were generated or analysed during the current study.
