# Supplementary material for: Immune biomarkers for head and neck cancer
Source: Cancer Immunol Immunother. 2025 Dec 18;75(1):6. doi: 10.1007/s00262-025-04233-7 (PMC12715102; doi:10.1007/s00262-025-04233-7)
Supplement: Supplementary file 1 — Supplementary file1 (DOCX 3858 KB) [file 262_2025_4233_MOESM1_ESM.docx]

**
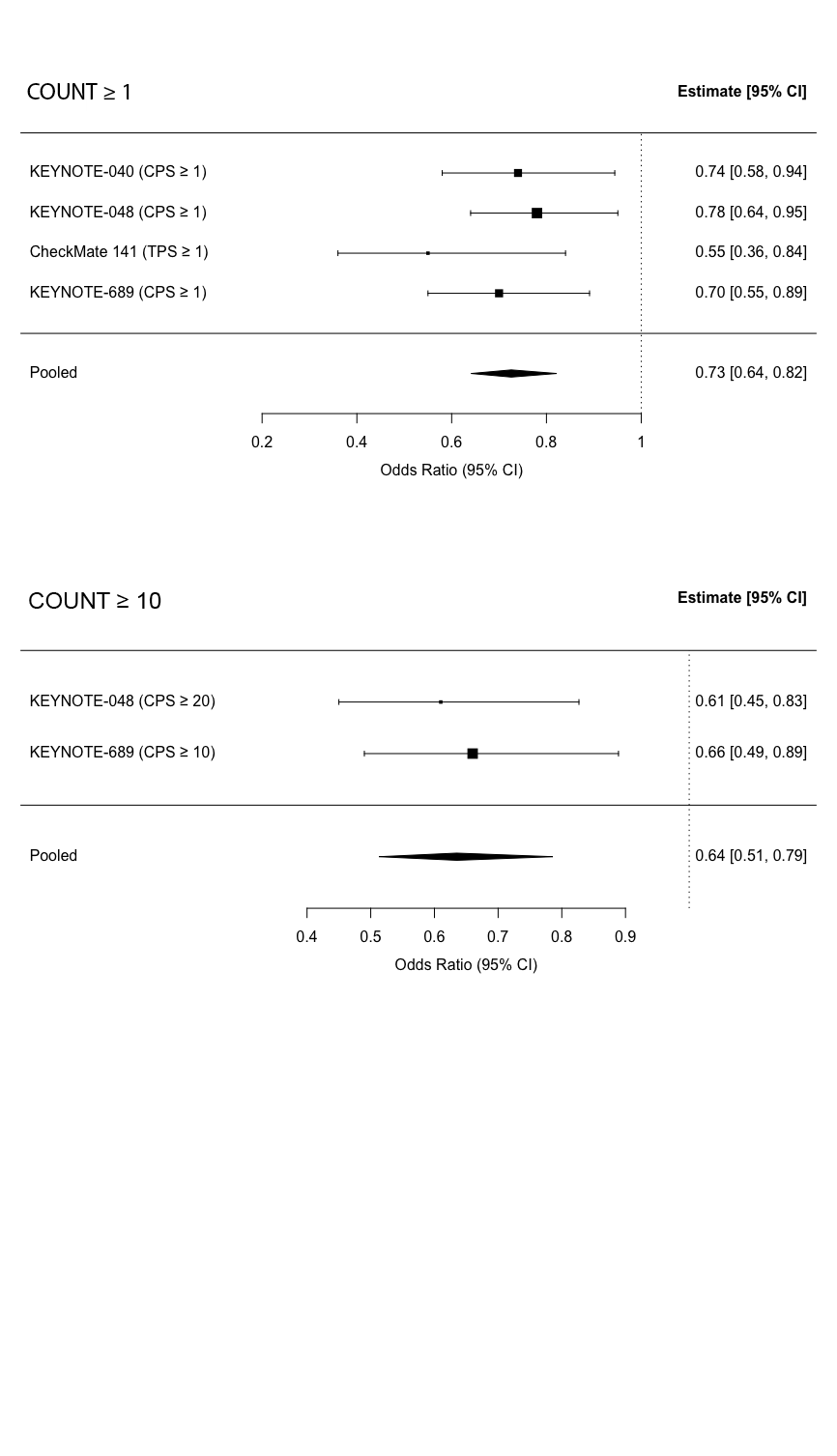
**

**Fig 3 Meta-analysis of proportion scores.** Three studies with proportion scores ≥ 1 were compared to two studies with CPS ≥ 10 to investigate any difference in predicting immunotherapy efficacy to improve survival of head and neck cancer patients.
